# Implant supracrestal complex conditioning through the “x technique” to achieve an enhanced muco-cervical imprint. Case reports

**DOI:** 10.21142/2523-2754-1302-2025-246

**Published:** 2025-05-16

**Authors:** Alberto Miselli, Ana Luisa Bernotti, Patricia Moreno-Garcés, Ioannis Vergoullis, Carlos Sánchez-Ramírez

**Affiliations:** 1 DDS, Specialist in Prosthodontics and Dental implants. Dentalmis Dental Center. Caracas, Venezuela. dentamlims@gmail.com DDS, Specialist in Prosthodontics and Dental implants. Dentalmis Dental Center Caracas Venezuela dentamlims@gmail.com; 2 DDS, Specialist in Dental Implants and Periodontics, Doctoral Student in Dentistry. Central of Venezuela University, Caracas, Venezuela. bernottianaluisa@gmail.com Universidad Central de Venezuela DDS, Specialist in Dental Implants and Periodontics, Doctoral Student in Dentistry Central of Venezuela University Caracas Venezuela bernottianaluisa@gmail.com; 3 DDS, Specialist in Orthodontics. Assistant Professor, Department of Radiology, Central of Venezuela University. Caracas, Venezuela. pattyrusia@gmail.com Universidad Central de Venezuela DDS, Specialist in Orthodontics. Assistant Professor, Department of Radiology Central of Venezuela University Caracas Venezuela pattyrusia@gmail.com; 4 DDS, MS. Diplomate, American Board of Periodontology. Diplomate & Fellow, ICOI. Visiting Clin. Assistant Professor. Department Periodontics, Louisiana State University (LSU), USA. drvergoullis@gmail.com Louisiana State University DDS, MS. Diplomate, American Board of Periodontology. Diplomate & Fellow, ICOI. Visiting Clin. Assistant Professor. Department Periodontics Louisiana State University (LSU) USA drvergoullis@gmail.com; 5 DDS. MSc. Oral Medicine. Assistant Professor, Department of Oral Pathology, Jose Antonio Páez University. Valencia, Venezuela. odcarlossanchez@gmail.com Universidad José Antonio Páez DDS. MSc. Oral Medicine. Assistant Professor, Department of Oral Pathology Jose Antonio Páez University Valencia Venezuela odcarlossanchez@gmail.com

**Keywords:** X technique, implant supracrestal complex, muco-cervical imprint, active oxygen, lactoferrin, técnica X, complejo supracrestal del implante, impronta muco-cervical, oxígeno activo, lactoferrina

## Abstract

In dentistry, predictable and successful implant treatments demand a comprehensive understanding of the biological mechanisms related to hard and soft tissue changes. The objective of this case reports is to describe in two patients, the conditioning of the implant supracrestal complex (ISC) through the "X Technique", aiming to achieve an enhanced muco-cervical imprint of an individualized emergence profile conformer, reproducible for the emergence profile of the final prosthetic restoration, safeguarding ISC health with the use of active oxygen/lactoferrin gel (blue®m) and ISC stability with the proposed surgical-prosthetic protocol. The technique involves oblique subperiosteal incisions in an "X" pattern with meticulous tissue manipulation to modify peri-implant mucosa phenotype, enhance the buccal contour, and stimulate papilla formation without resorting to additional mucosal grafts. Clinical significance: The technique described offers a promising approach in obtaining a predictable and reproducible muco-cervical imprint or footprint of the prosthetic emergence profile framed within the ISC. Offering to achieve key parameters such as supracrestal tissue height (STH), ISC integrity, muco-cervical imprint stability and implant mucosa thickness (MT) displaying stability. This underscores the effectiveness of the technique in ensuring the sustained health and aesthetics of peri-implant tissues. Further long-term studies are needed to quantify the benefits of this technique in increasing keratinized tissue width (KTW) and MT effectively.

## RESUMEN

En odontología, los tratamientos predecibles y exitosos con implantes exigen una comprensión integral de los mecanismos biológicos relacionados con los cambios en los tejidos duros y blandos. El objetivo de este reporte de casos es describir, en dos pacientes, el acondicionamiento del complejo supracrestal del implante (CSI ) a través de la técnica X, con el objetivo de lograr una impronta o huella muco-cervical mejorada del conformador de perfil de emergencia individualizado, reproducible para el perfil de emergencia de la restauración protésica final. Esto con el fin de salvaguardar la salud del CSI con el uso de oxígeno activo/lactoferrina en gel (blue®m) y la estabilidad del CSI con el protocolo quirúrgico-protésico propuesto. La técnica consiste en incisiones subperiósticas oblicuas en un patrón de "X" con una meticulosa manipulación del tejido para modificar el fenotipo de la mucosa periimplantaria, mejorar el contorno bucal y estimular la formación de papilas sin recurrir a injertos mucosos adicionales. Importancia clínica: La técnica descrita ofrece un enfoque prometedor en la obtención predecible y reproducible de una impronta o huella muco-cervical del perfil de emergencia protésico enmarcado dentro del CSI. Permite alcanzar parámetros clave como la altura de los tejidos supracrestales (STH), la integridad del CSI, la estabilidad de la impronta muco-cervical y el grosor de la mucosa del implante (MT), y muestra estabilidad. Esto subraya la eficacia de la técnica para garantizar la salud y estética sostenidas de los tejidos periimplantarios. Se necesitan estudios a largo plazo para cuantificar los beneficios de esta técnica en el aumento efectivo de la banda de tejido queratinizado (KTW) y el MT.

## INTRODUCTION

Creating effective treatments in implant dentistry requires a deep comprehension of the biological mechanisms governing changes in the size of both hard and soft tissues. This understanding should encompass surgical methodologies utilized and the interplay between implant surfaces/prosthetic elements and the adjacent tissues ^1^. Chappuis et al., considered that in areas with thick phenotypes, the correct positioning of implant-supported rehabilitation is facilitated. This is because these sites tend to exhibit limited bone resorption rates, allowing the peri-implant mucosa to be supported by an adequate three-dimensional bone volume ^2^. It has been thought that soft tissue augmentation around implants centered in increasing keratinized mucosa width (KMW) as well as mucosa thickness (MT) leads to supracrestal tissue height (STH) health and stability ^3^. The need to increase MT is often associated with preventing peri-implant bone loss and improving aesthetic outcomes ^4^. It's not surprising that thick mucosal tissue often results in improved aesthetics, as it can achieve better tissue contour, leading to more favorable outcomes ^5^. Additionally, thick mucosal tissue aids in creating papilla after the prosthesis is delivered ^6^. Rathi et al., ^7^ reported a study in humans, that repeated abutment replacements may insult mechanically the mucosa barrier, that might initiate other toxic irritants and bacteria into the mucosal‑implant interphase that may affect the strength of the tissues around implants. The development of the “definitive abutment,” might minimize the chances of peri‑implant soft and hard tissue loss. 

Emerging evidence implies significant interrelations between the condition of the peri-implant tissues and the implant-abutment-prosthesis complex. Prosthetic-driven implant placement is a prerequisite for a proper implant supracrestal complex (ISC) design, which in turn can indirectly influence the structure and dimensions of the peri-implant soft tissues. Design features of the implant-prosthesis-abutment complex, such as the emergence profile, emergence angle, and cervical margin, as well as the design of the implant-abutment and abutment-prosthesis junctions and their locations in relation to ISC, can have a significant impact on the maintenance of a stable and healthy peri-implant tissues in the long term ^8^. Conditioning of the soft tissue phenotype around the implant can be undertaken at various stages of implant treatment without resulting in significant reductions of KMW or MT ^9^. Furthermore, this procedure can be carried out concurrently with prosthetic procedures, eliminating the need for additional surgeries ^10^. 

Hence, the main of this study was devised to describe the ISC conditioning through the “X Technique” to achieve an improved muco-cervical imprint of an individualized emergence profile conformer, that will remain unaffected by the emergence profile of the final prosthetic restoration, thus preserving the health and stability of the ISC. Therefore, among the objectives of this study, it will be describing the ¨X technique¨ as a modifier of the peri-implant phenotype, coupled with the use of the individualized emergence profile conformer (Cervico® VP Innovato Holdings Ltd, Cyprus). Cleaning the biomaterial surface and implant features with the use of active oxygen/ lactoferrin gel (blue® m) as well as disinfecting the surgical area ^11^.

## PATIENT SELECTION 

The present study included 2 patients who had at least one implant placed. Patients in the study met the inclusion criteria: Patients treated with at least one crestal or subcrestal implant, with a minimum vertical mucosal thickness of 3 mm, requiring unitary crown restorations. It was designed following the principles of the Declaration of Helsinki ^12^. Likewise, informed consent was used, which stated that, for research, the identity of the patients will be preserved; all study participants they were treated under the principles of beneficence, non-maleficence, and justice. 

Patients with uncontrolled systemic disease, such as hypertension, diabetes, untreated periodontitis, and smokers (more than 10 cigarettes/day) were excluded. All surgeries were performed by a specialist in implantology or periodontics.

## STUDY DESIGN 

The study involved describing the "X Technique” for conditioning the ISC to achieve an improved muco-cervical imprint or footprint of an individualized emergence profile conformer (Cervico® VP Innovato Holdings Ltd, Cyprus). This technique was performed after the completion of implant osseointegration and soft tissues fully healed around the stock healing abutments. A slow-release active oxygen/lactoferrin gel (blue® m) was applied in all cases for a minimum of 2 minutes to prevent bacterial proliferation and chemical contamination, decontaminate prosthetic components, and promote wound healing ^11^. Post-operative and oral hygiene instructions were provided to all patients (analgesic: Ibuprofen 200 mg twice a day for 2 days). Follow-ups were done.

## DESCRIPTION OF THE TECHNIQUE

**Figure 1:**
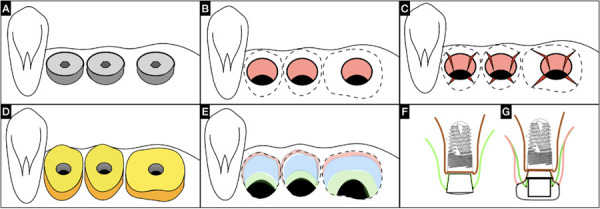
Illustration of the ISC conditioning through the “X Technique”: A) Soft tissues fully healed around the stock healing abutments. B) Removal of healing abutments, revealing the implant platforms through the mucosal tunnel of the ISC. C) ¨X Technique” incisions, subperiosteal releasing oblique incisions (two palatal/lingual and two buccal). D) Individualized emergence profiles conformers Cervico® VPI in position. E) ISC conditioned zones. F) Side view of the implant with stock healing abutment showing mucosa thickness in green line. G) Side view of the implant with individualized emergence profile conformer Cervico® VPI after ¨X Technique” showing with pink line, increased mucosal thickness.

The ¨X Technique¨ is a second stage surgery done after implant osseointegration and soft tissues fully healed around the stock healing abutments (Figure 1A). The ISC is established through the mucosa tunnel created with the healing screw or stock healing abutment (Figure1B). Subsequently, after local anesthesia administration, the “X Technique” may be performed. The “X Technique” involves four (4) oblique subperiosteal incisions (two palatal/lingual and two buccally), in an "X" pattern with meticulous tissue manipulation to modify peri-implant mucosa phenotype, enhance the buccal contour, and stimulate papilla formation, with a wider base at the apical zone and vertex coronally near the cervical mucosa margin (Figure 1C). The ¨X Technique¨ is known as a ISC collagen fibers release technique that allows to accomplish an enhanced muco-cervical imprint or footprint of the individualized emergence profile conformer (Cervico® VP Innovato Holdings Ltd, Cyprus) or final prosthetic emergence profile (Figure 1C-1D). Avoid excessive tissue compression or ischemia, as these can disrupt the healing process and alter tissue dimensions inappropriately. Flap suturing is not required. Preservation of the proximal tissues, mesial and distal, should be achieved to facilitate horizontal displacement of the flaps towards the periphery through slight compression made with the screwed individualized emergence profile conformer (Cervico® VP Innovato Holdings Ltd, Cyprus), in correspondence with the tooth emergence profile cervical diameter to be restored (Figure 1D). Resulting in ISC conditioned zones (Figure 1E) avoiding resorting to additional mucosal grafts. The technique described offers a promising approach in obtaining a predictable and reproducible muco-cervical imprint or footprint of the prosthetic emergence profile framed within the ISC. (Figure 1E).

## CLINICAL CASES

### CLINICAL CASE 1

**Figure 2:**
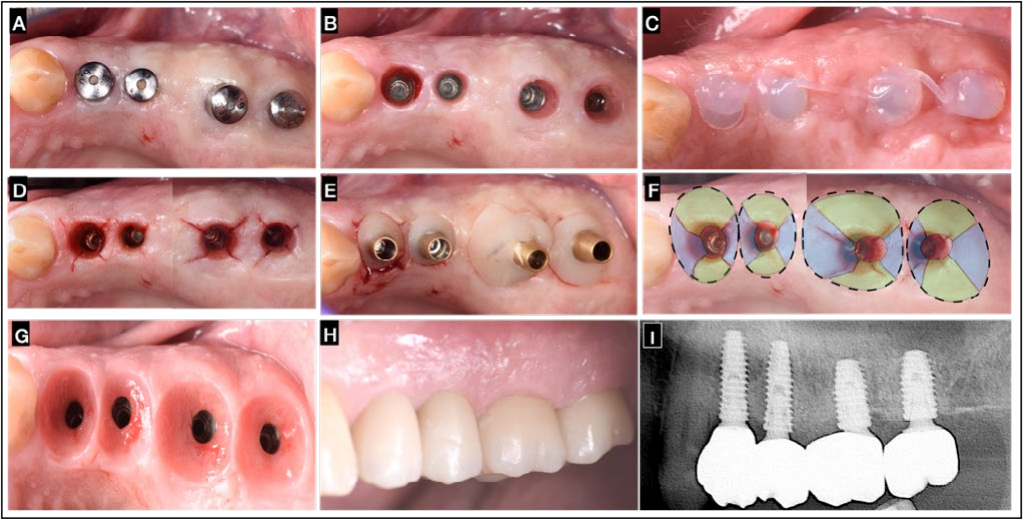
“X Technique” in Adjacent Implants: A) Soft tissues fully healed around the stock healing abutments. B) Removal of healing abutments, revealing the implant platforms through the mucosal tunnel of the ISC. C)blue®m gel applied for 2min. D) Subperiosteal “X Technique” incisions on the ISC at all four implant sites. E) Placement of the individualized emergence profile conformer (Cervico® VPI) on all 4 implants. F) Tissue conditioning and areas of compression to the periphery at buccal, palatal and interproximal areas. G) Muco-cervical imprint or footprint within the ISC of the crown’s emergence profile after 6 months of healing. H) 1 year follow up individually cemented/screwed multilayer zirconia crowns rehabilitation at 2-4,2-5,2-6,2-7 zones, note the formation of well-defined papillae, mucosa zeniths and buccal mucosa volume. I) Radiographic control at 1 year after final rehabilitation delivery.

A 65-year-old male patient who attended the clinic for implant rehabilitation, whose main reason: ¨ Improve my smile and eat better ¨. The intraoral examination revealed at the II quadrant the presence of four implants at the posterior edentulous area, after implant osseointegration with soft tissues fully healed around stock healing abutments (Figure 2A). Removal of healing abutments, revealed the implant platforms through the mucosal tunnel of the ISC, created during tissue healing (Figure 2B). A slow-release active oxygen/lactoferrin gel (blue® m) was applied, for at least 2 minutes, to prevent bacterial proliferation and chemical contamination, safeguarding ISC health and stability during wound healing ^11^ (figure 2C). The second stage surgery, known as a ISC collagen fibers release technique, the ¨X Technique was conducted under local anesthesia. The technique involves oblique subperiosteal incisions in an "X" pattern (two palatal/lingual and two buccal) with meticulous tissue manipulation to modify peri-implant mucosa phenotype, enhance the buccal contour, and stimulate papilla formation assisted by the progressive compression of an Individualized emergence profiles conformers (Cervico® VPI) accoupled to each of the four implants in congruence with the prosthetic emergence profile of each tooth to be rehabilitated (Figure 2D), thus avoiding resorting to additional mucosal grafts. Conditioning the tissue with areas that can be easily displaced through compression toward the implant periphery in the buccal, palatal, and interproximal regions (Figure 2E). No flap suturing is required. The ¨X Technique¨ allows to accomplish within the ISC an enhanced muco-cervical imprint or footprint of the individualized emergence profile conformer or final prosthetic emergence profile (Figure 2F - 2G). With this type of guided tissue healing achieved through the Cervico® VPI, individually cemented/screwed multilayer zirconia crowns can be delivered after 30 days. Post-operative and oral hygiene instructions (analgesic: Ibuprofen 200 mg twice a day for 2 days), was given to the patient. This process ensured that new prosthetic restoration did not disturb the soft tissues, allowing for the formation of well-defined papillae and zeniths as it can be noticed at 6 months and one year follow up (figure 2G-2H). Additionally, it supports the gradual establishment of peri-implant bone over time, 1 year follow up (Figure 2I).

### CLINICAL CASE 2

**Figure 3:**
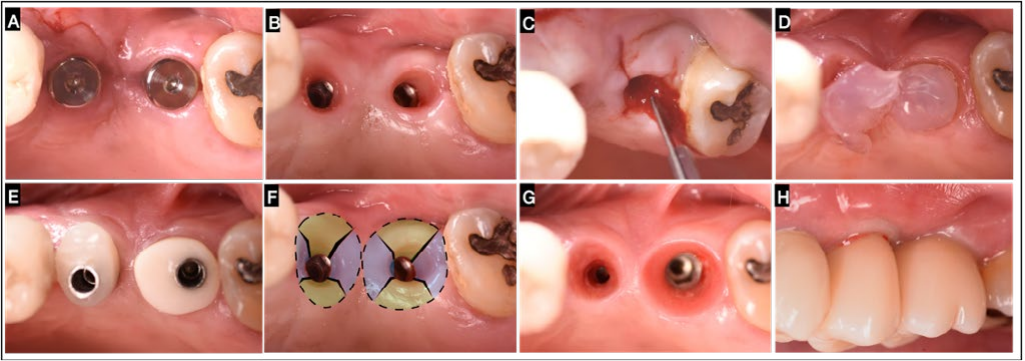
“X Technique” in Adjacent Implants: A) Soft tissues fully healed around the stock healing abutments at 2-5,2-6 implant zone. B) Removal of healing abutments, revealing the implant platforms through the mucosal tunnel of the ISC. C) Subperiosteal “X Technique” incisions on the ISC at implant sites. D) blue®m gel appliance for two minutes. E) Placement of the individualized emergence profile conformer (Cervico® VPI) on implants. F) Tissue conditioning and areas of compression to the periphery at buccal, palatal and interproximal areas. G) Muco-cervical imprint of the individualized emergence profile conformer after 30 days of healing. H) Day "0" delivery of individually cemented/screwed multilayer zirconia crowns.

A 62-year-old female patient who attended the clinic for implant rehabilitation, whose main reason: ¨I want to recover all my teeth¨. The intraoral examination revealed at the II quadrant the presence of two implants at the 2-5 and 2-6 zone, after implant osseointegration with soft tissues fully healed around stock healing abutments (Figure 3A). Removal of healing abutments, revealing the implant platforms through the mucosal tunnel of the ISC (Figure 3B). Subperiosteal incisions of the “X technique” in the ISC were performed (Figure 3C) Clot chambers are formed in the incision’s areas, promoting increased mucosa volume and peripheral sealing (Figure 3C), a slow-release active oxygen/lactoferrin gel (blue®m) was applied in the area (Figure 3D). The individualized emergence profile conformers (Cervico® VP Innovato Holdings Ltd, Cyprus) were screwed on the implants (Figure 3E). With slight compression, tissue conditioning and flap distension towards the periphery of the buccal, palatal, and proximal areas were achieved, resulting in a more prominent buccal contour along with the formation of inter-implant papilla (Figure 3F). The muco-cervical imprint or footprint of the emergence profile after 30 days of healing was observed to be stable and healthy in each implant area (Figure 3G), facilitating the impression-taking process, capturing not only the implant position but also the muco-cervical imprint left on the ISC by the individualized emergence profile conformer (Cervico® VP Innovato Holdings Ltd, Cyprus). This allowed the delivery of a zirconia crown with identical characteristics to the previously established emergence profile contour, thereby preventing soft tissue alterations that could compromise the health and stability of the ISC in conjunction with marginal bone loss (Figure 3G, 3H). Highlighting the papillae formation, increased buccal contour and mucosa architecture achieved with the proper positioning of the prosthetic zenith (Figure 3G, 3H).

**Figure 4:**
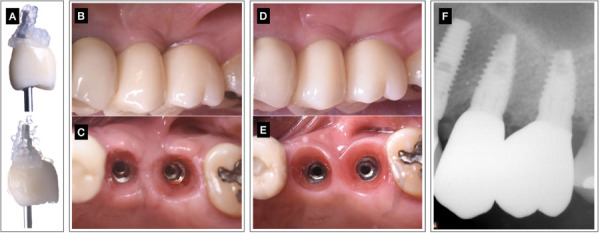
A) Removal of individually cemented/screwed multilayer zirconia crowns (2-5 & 2-6) and blue®m gel appliance before re-inserting them in each of the follow’s controls. B) 6 month follow up of individually cemented/screwed multilayer zirconia crowns rehabilitation at 2-5 and 2-6, buccal view. Observe peri-implant mucosa volume and papillae formation. No black triangle presence. C) Muco-cervical imprint of the crown emergence profile at 2-5, 2-6-zone after 6 months follow up of tissue healing. D) 1 year follow up of individually cemented/screwed multilayer zirconia crowns rehabilitation at 2-5 and 2-6, buccal view. Observe buccal volume improvement. E) Muco-cervical imprint or footprint within ISC of zirconia crowns emergence profile at 2-5, 2-6-zone after 1 year of healing. F) Radiographic control of the definitive implant-crown rehabilitation at 1 year.

The findings of clinical case 2, demonstrated after 30 days, 6 months and 1 year follow up, that the implementation of the "X Technique" allows for significant improvements in peri-implant mucosal contour, the preservation of proximal tissue, and the successful formation of inter-implant papilla (Figure 3G,4C,4E). These outcomes are achieved without requiring additional mucosal grafts, by performing incisions in an "X" pattern with meticulous tissue manipulation especially when combined with the use of an individualized emergence profile conformer (Cervico® VP Innovato Holdings Ltd, Cyprus) (Figure 3E). This approach effectively addressed buccal and interproximal soft tissue as it promoted black triangles closure (Figure 3H). 

Within the ISC a muco-cervical imprint or footprint was precisely crafted to define the prosthetic emergence profile, ensuring proper mucosal architecture and optimal positioning of the mucosal zenith. At the 30-day follow-up, the results remained stable, allowing the placement of a zirconia crown with identical characteristics to the pre-established emergence profile. This careful alignment prevented any alterations to the soft tissues that could compromise ISC health or lead to marginal bone loss. The study emphasizes the importance of maintaining the three-dimensional implant position to respect the ISC in relation to the crown’s cervical margin and prosthetic zenith, as it can be seen 6 months (Figure 4B,4C) and 1 year follow up (Figure 4D,4E). Additionally, the use of active oxygen/lactoferrin gel (blue®m) on the ISC and biomaterial surfaces created ideal disinfection conditions, further supporting a predictable biological seal (Figure 3D). There was no uneven texture or scar formation, and healing proceeded with minimal interruption. Finally, it is a non-technique-sensitive procedure. In these cases, all patients reported high levels of satisfaction with the procedures and the active oxygen/lactoferrin gel (blue®m) products utilized during their healing process.

At the one-year follow-up (figure 4D,4E), key parameters such as soft tissue health (STH), ISC integrity, muco-cervical imprint stability, implant mucosa thickness (MT) and peri-implant tissue health demonstrated long-term stability. This underscores the effectiveness of the technique in ensuring the sustained health and aesthetics of peri-implant tissues.

## DISCUSSION

Following implant placement surgery, achieving successful osseointegration is paramount. However, it is acknowledged that the peri-implant mucosal phenotype also plays a significant role in obtaining satisfactory biological and prosthetic aesthetic outcomes. Peri-implant plastic surgery enhances structures around implants using tissue engineering techniques, including improving bone and soft tissue, precision in implant placement, and prosthetic restoration quality ^13^^,^^14^. 

Prosthetic-driven implant placement is a prerequisite for proper ISC design, which in turn can indirectly influence the structure and dimensions of the peri-implant soft tissues. Design features of the implant-prosthesis-abutment complex, such as the emergence profile, emergence angle, and cervical margin, as well as the design of the implant-abutment and abutment-prosthesis junctions and their locations in relation to the tissues of the ISC, can have a significant impact on the maintenance of stable and healthy peri-implant tissues in the long term ^8^. The peri-implant soft tissue phenotype (PSP) includes various aspects such as KMW, MT, and STH. Many methods for increasing soft tissue volume around dental implants have been studied ^4^.

A recent systematic review concluded that MT gain may also promote greater stability of interproximal marginal bone levels ^15^. The modification of the PSP through effective surgical techniques between implant areas can be beneficial for peri-implant health. Combined with appropriate prosthetics emergence profile, allows for obtaining a muco-cervical imprint, facilitating biological seal and interproximal papillae. This contributes in achieving healthy, functional, and aesthetic outcomes in implant areas ^4^.

The introduction of new, improved, or modified periodontal plastic surgical techniques addresses nearly all mucogingival challenges, except for the loss of papillae. Therefore, it is of critical importance to develop and define aesthetic guidelines for treatment of the ISC. In these guidelines, the aesthetic analysis of a treatment is divided into an evaluation of the mucogingival and that of the tooth or prosthetic structure. Correction of mucogingival discrepancies is a prerequisite for aesthetic success in dental implant treatment ^16^. 

Palacci et al. ^14^, detail a surgical technique involving partial grafting of the flap during implant uncovering. While effective, this method demands both technical skill and adequate mucosal thickness. This study found that employing the "X Technique” alongside the individualized emergence profile conformer ^17^ (Cervico® VP Innovato Holdings Ltd, Cyprus) during or after implant uncovering enhances peri-implant mucosal volume or thickness. 

This creates a favorable mucosal architecture for achieving ideal aesthetic outcomes around the definitive crown. Moreover, it stimulates papillae formation between implants and adjacent teeth being flap suturing not required, in most of the clinical cases. The "X" incisions pattern divides the peri-implant mucosal tissue into four segments. With slight compression from the individualized emergence profile conformer, these segments are displaced from the implant towards the periphery, augmenting buccal mucosal contour and proximal soft tissue volume in both height and width. Papillae vascularity is achieved due the “X Technique” leads the incisions to be perform out of proximal area, avoiding the repeated supply disruption of the vascular anastomoses crossing the alveolar ridge ^18^.

However, Bichacho et al. ^13^^)^ recommends to de-epithelialize the mucosa tunnel walls in their inner aspect, with a water-cooled high-speed coarse diamond bur, to reach the vascularized connective tissue. Repeated vascular disruption triggers scar tissue formation, characterized by dense, rigid, and poorly vascularized tissue, complicating molding efforts. Reducing scar tissue formation facilitates reshaping of mucosa tissue under light pressure, crucial for maintaining or restoring papillae integrity ^18^^,^^19^.

Achieving and preserving papillary tissue integrity around implants requires meticulous planning and execution, as demonstrated in this study, specific incision designs and blood clot maintenance assisted with the Cervico® VPI, to achieve better healing ^20^, and soft tissue management techniques play a crucial role in regrowing the proximal papillae. Preserving adjacent papillary blood supply, bone integrity, and minimizing scar tissue formation are key factors for successful outcomes ^21^^,^^22^. 

The volume of the ISC significantly influences the creation of the ideal emergence profile around the dental implants. It is recommended that the deepest part of the emergence profile have a divergence of less than 30° to avoid marginal bone loss and achieve optimal aesthetic and functional outcomes ^23^. An ongoing challenge for implant restorative dentists is achieving an aesthetic transition from the smaller implant diameter to the prosthetic restoration, resembling the size of a natural tooth. The appearance of surrounding soft tissue is crucial, necessitating guided prosthetic work in the gingival architecture based on cervical contouring concepts and the location of the contact area to modify soft tissue topography. Nonsurgical restorative techniques, such as using an individualized emergence profile conformer like Cervico® VPI, helps manipulate the soft tissue around the cervical aspect of the implant into a more favorable contour. This ensures complete sealing of the ISC tunnel, preventing physical interferences and bacterial and chemical contamination of the tissue on the muco-cervical imprint. Additionally, treating with active oxygen/Lactoferrin gel (blue®m) to decontaminate biomaterial surface and wound disinfection, helps to prevent apical migration of the junctional epithelium due to a challenge from biofilm-induced chronic inflammation and marginal bone loss ^8^^,^^3^^,^^17^^,^^24^.

## CONCLUSIONS

Enhancing soft tissues around dental implants - Crowns is crucial for meeting patients' biological, functional, and aesthetic requirements. Emphasizing second-stage surgery over merely placing healing abutments is essential. Adjusting the periodontal phenotype with the “X Technique” during this phase can enhance tissue contour, thickness, and overall quality. Further long-term studies are needed to quantify the benefits of this technique in increasing KTW and MT effectively. Incorporating the “X Technique” into routine implant procedures has the potential to significantly improve patient outcomes and satisfaction.

## Financing

This research work was self-financed
